# The long-term observation of the rotation of implantable collamer lens as the management of high postoperative vault

**DOI:** 10.3389/fmed.2023.1104047

**Published:** 2023-02-23

**Authors:** Yang Jiang, Yan Luo, Ying Li, Thomas Lu

**Affiliations:** ^1^Department of Ophthalmology, Peking Union Medical College Hospital, Chinese Academy of Medical Sciences, Beijing, China; ^2^School of Medicine, University of New South Wales, Kensington, NSW, Australia

**Keywords:** implantable collamer lens, high vault, rotation, secondary complications, foldable intraocular lens

## Abstract

**Purpose:**

This study aimed to describe the effectiveness and stability of implantable collamer lens (ICL) rotation in reducing high postoperative vault.

**Setting:**

This study was conducted in the Peking Union Medical College Hospital, Chinese Academy of Medical Sciences.

**Design:**

This is a retrospective case series.

**Methods:**

Twenty-two eyes from 22 patients who had ICL (V4c EVO) implantation with high postoperative vault (>=750 μm) were recruited for our study. All the lenses were rotated from a horizontal to an oblique position. The vault, SSA, AOD500, AOD750, TISA500, and TISA750 at 180° and 0° were measured pre-rotation, 1-week post-rotation, and in the at-least 1-year-follow-up.

**Results:**

Twenty female patients and two male patients were recruited, with a mean age of 28.68 ± 6.08 years. The mean vault had declined significantly from 951.81 ± 154.26 μm pre-rotation to 772.27 ± 119.40 μm 1 week post-rotation (*p* < 0.001). The SSA, AOD500, AOD750, TISA500, and TISA750 at 180° and 0° pre-rotation and 1-week post-rotation were 30.40 ± 7.91° and 45.14 ± 6.75°, 32.37 ± 7.48° and 46.23 ± 6.39°, 303.27 ± 87.99 and 522.45 ± 122.16 μm, 323.81 ± 89.15 and 536.13 ± 121.66 μm, 387.95 ± 99.43 and 630.81 ± 133.59 μm, 435.68 ± 106.72 and 643.36 ± 132.82 μm, 0.109 ± 0.034 and 0.202 ± 0.053 mm^2^, 0.123 ± 0.034 and 0.212 ± 0.051 mm^2^, 0.194 ± 0.056 and 0.345 ± 0.083 mm^2^, and 0.216 ± 0.055 and 0.358 ± 0.079 mm^2^ (all *p* < 0.001). The mean vault value had changed from 747.50 ± 116.07 μm 1-week post-rotation to 586.87 ± 132.65 μm in the 1-year follow-up. However, the SSA, AOD500, AOD750, TISA500, and TISA750 at 180° and 0° had remained stable (*p* > 0.05).

**Conclusion:**

Non-toric ICL rotation is a novel and effective technique in the treatment of high postoperative vault. Our results are more robust given the extended period of follow-up.

## Background

The Implantable Collamer Lens (ICL; STAAR Surgical, Monrovia, CA) is a foldable intraocular lens (IOL) that is increasingly used to correct myopia. The procedure is relatively safe and effective (1). The ICL is positioned between the iris and the crystalline lens, with the haptics footplates resting on the ciliary sulcus. The distance between the ICL and the crystalline lens is referred to as the vault.

An optimal postoperative vault is important to prevent secondary complications. The postoperative high vault is associated with elevated intraocular pressures, glaucoma, endothelial cell loss, and pupil distortion (1). The natural curvature of the lens and the relationship between the size of the ICL and the width of the ciliary sulcus are all parts in the determination of vault size.

The shape of the ciliary sulcus has previously been demonstrated to be vertically oval (2). We hypothesize that the rotation of ICL from a horizontal position to an oblique position will reduce vault size. This may be an appropriate alternative to ICL exchange, which has been traditionally utilized for postoperative high vault cases (3). One successful reduction of the high postoperative vault by the means of ICL rotation has been described in a case report (4). Zaldivar et al. have reported their satisfactory results of ICL rotation under the guidance of intraoperative OCT (5). Our study is the first case series to demonstrate the effectiveness of this novel surgical procedure in the treatment of high postoperative vault without the use of intra-operative OCT.

## Methods

### Patient selection

The recruited patients should meet all of the following inclusion criteria: (1) non-toric ICL V4c lenses implanted; (2) successful operation without intra-operative adverse events; and (3) high post-operative vault (>=750 μm) following ICL implantation. The aim of this procedure is to prevent complications related to high vault rather than treat the complications. Therefore, the rotation would be practiced within 1 week after the surgery. The study adhered to the tenets of the Declaration of Helsinki. Retrospective data collection was approved by the Institutional Ethics Committee of the Chinese Academy of Medical Sciences, Peking Union Medical College Hospital.

### Surgical technique

Topical anesthesia was used in all the surgeries, which were carried out by the same experienced surgeon (LY). A 3-mm clear corneal incision was performed at the horizontal position in the temporal side of the patient’s eye. Visco-elastic was injected into the anterior chamber. A spatula was used to carefully rotate the ICL by contacting the haptic footplates. ICL was rotated to 45 degrees oblique. The corneal incision was self-sealing after the removal of the visco-elastic.

### Study outcome parameters

All data were recorded pre-rotation, 1-week post-rotation, and in the 1-year follow-up. The scleral spur angle (SSA), angle opening distance 500 (AOD500), angle opening distance 750 (AOD750), trabecular-iris angle 500 (TISA500), and trabecular-iris angle 750 (TISA750) at 180° and 0° were measured by OCT (Zeiss Company, Germany). Descriptive statistics were used to quantitatively summarize data using mean values, standard deviations, and ranges. The paired *t*-test was utilized to determine the statistical significance of the change of paired data. A *p*-value <0.05 was considered statistically significant. Statistical analysis was undertaken in SPSS (version 20.0, IBM SPSS, Inc).

## Results

### Descriptive statistics of the sample

Our case series recruited 22 eyes, from 21 patients (20 female patients and 2 male patients), with a mean age of 28.68 ± 6.08 years. The preoperative spherical equivalent was −9.63 ± 3.13 D. The preoperative anterior chamber depth (ACD) was 3.29 ± 0.26 mm. The White-to-White (W to W) was 11.48 ± 0.43 mm. The ICL power was-11.02 ± 3.12 D (Supplementary Table 1).

### The effectiveness of rotation

**Figure 1:**
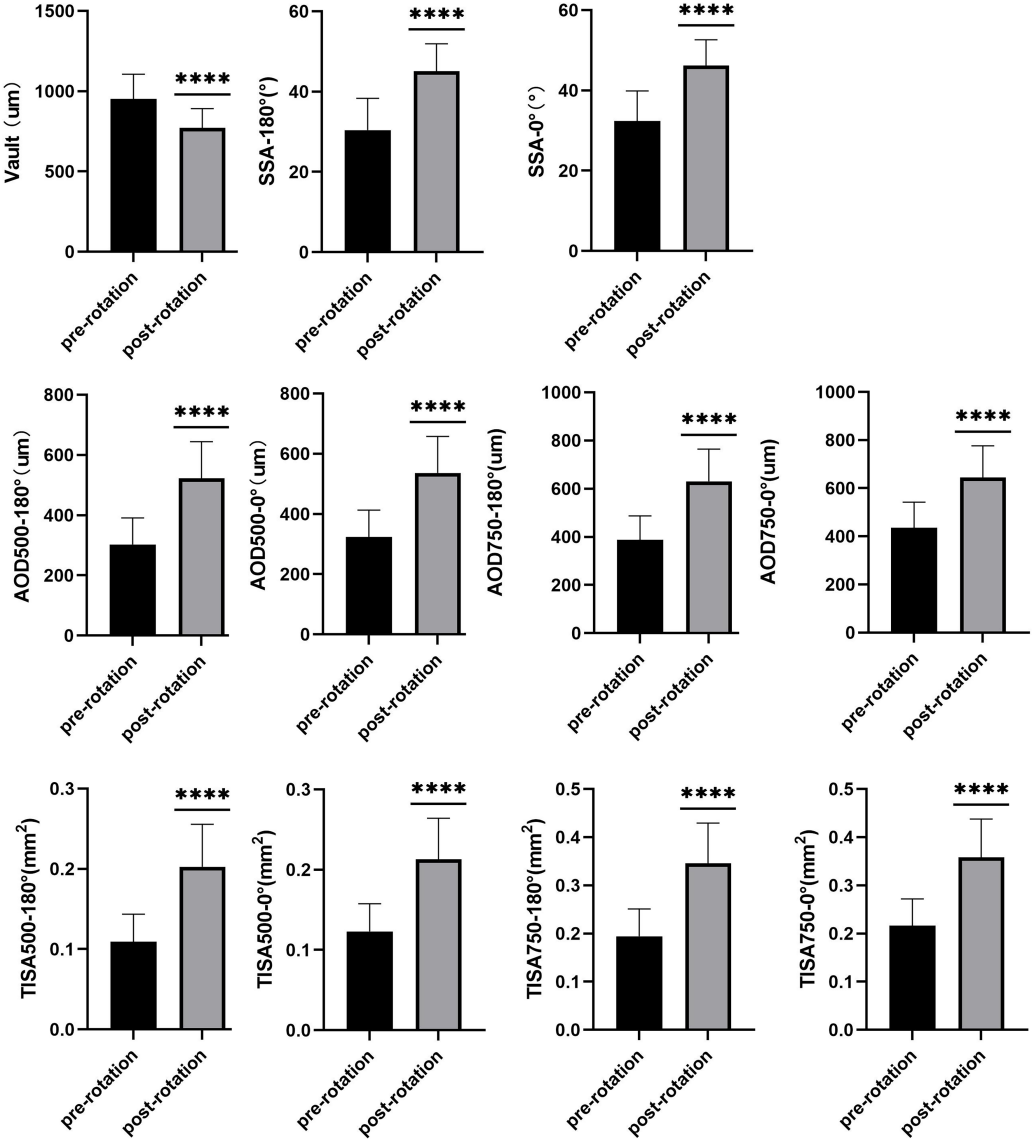
The mean vault value had declined significantly 1-week post-rotation (*p* < 0.001). The SSA, AOD500, AOD750, TISA500, and TISA750 at 180° and 0° had increased significantly accordingly (all *p* < 0.001). **** means that the *p* < 0.001.

**Figure 2:**
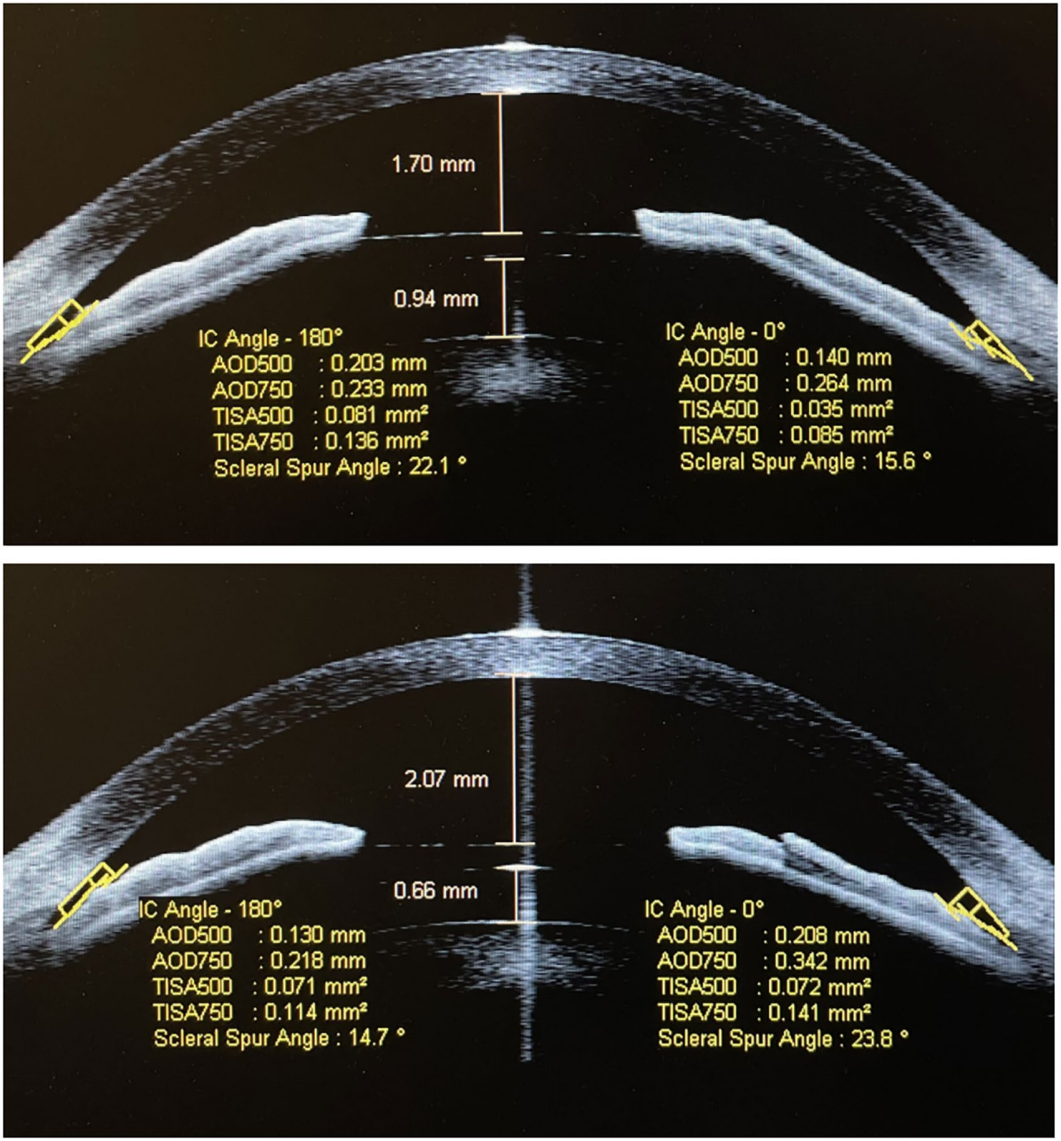
Anterior segment OCT image shows that the implantable collamer lens (ICL) vault was 940 μm and the anterior chamber depth was 1,700 μm before rotation **(A)** in patient A; 1 week after the rotation, the ICL vault was 660 μm and the anterior chamber depth was 2,070 μm **(B)**.

**Figure 3:**
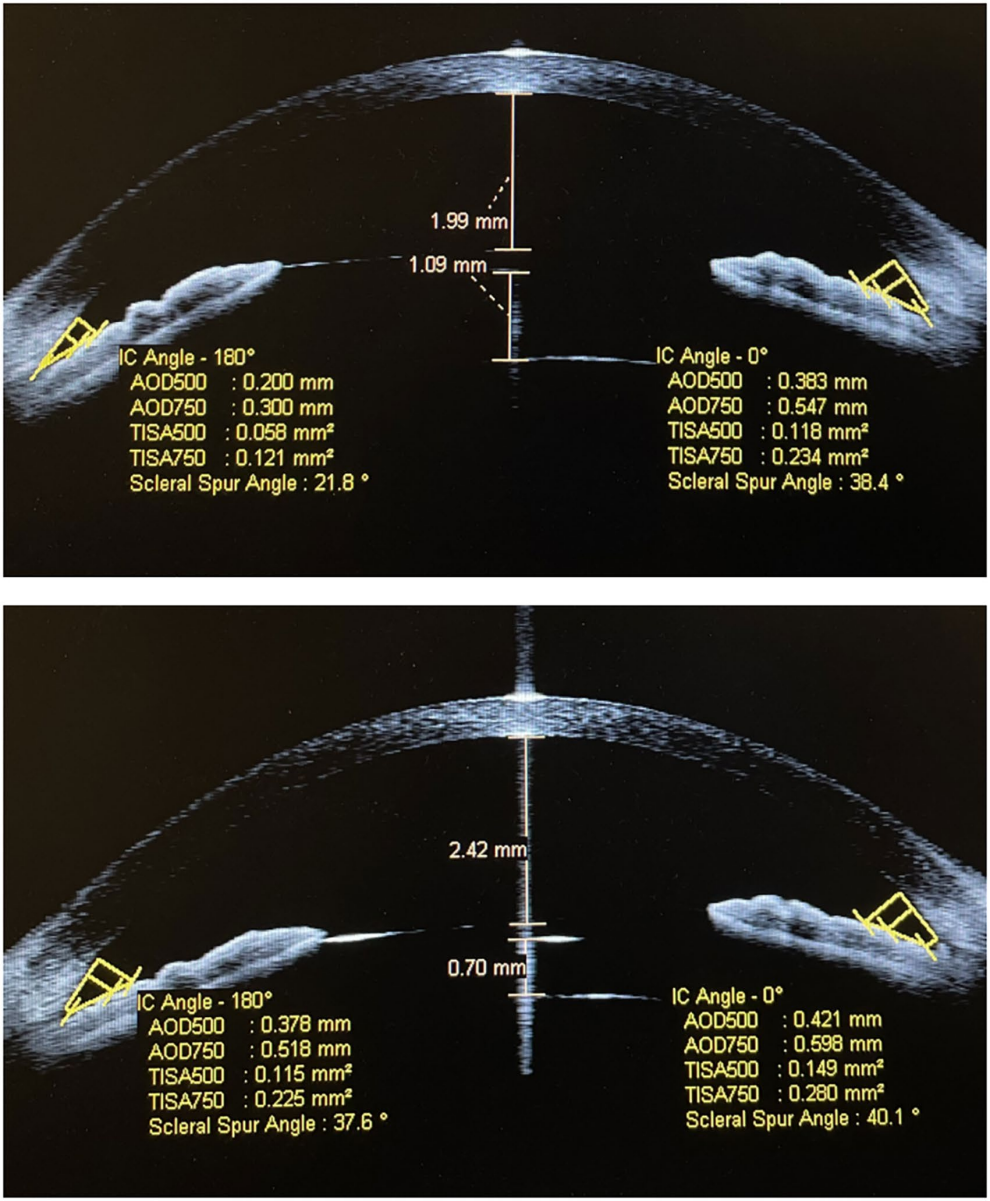
Anterior segment OCT image shows that the ICL vault was 1,090 μm and the anterior chamber depth was 1,990 μm before rotation **(A)** in patient B; 1 week after the rotation, the ICL vault was 700 μm and the anterior chamber depth was 2,420 μm **(B)**.

The mean vault value had declined significantly from 951.81 ± 154.26 μm pre-rotation to 772.27 ± 119.40 μm 1-week post-rotation (*p* < 0.001). The SSA, AOD500, AOD750, TISA500, and TISA750 at 180° and 0° pre-rotation and 1-week post-rotation were 30.40 ± 7.91° and 45.14 ± 6.75°, 32.37 ± 7.48° and 46.23 ± 6.39°, 303.27 ± 87.99 and 522.45 ± 122.16 μm, 323.81 ± 89.15 and 536.13 ± 121.66 μm, 387.95 ± 99.43 and 630.81 ± 133.59 μm, 435.68 ± 106.72 and 643.36 ± 132.82 μm, 0.109 ± 0.034 and 0.202 ± 0.053 mm^2^, 0.123 ± 0.034 and 0.212 ± 0.051 mm^2^, 0.194 ± 0.056 and 0.345 ± 0.083 mm^2^, and 0.216 ± 0.055 and 0.358 ± 0.079 mm^2^ (all *p* < 0.001; Supplementary Table 2; Figure 1). Anterior segment OCT image shows that the vault had declined significantly 1-week post-rotation, and the SSA, AOD500, AOD750, TISA500, and TISA750 at 180° and 0° had increased significantly accordingly (Figures 2, 3).

### The safety and stability of rotation

**Figure 4:**
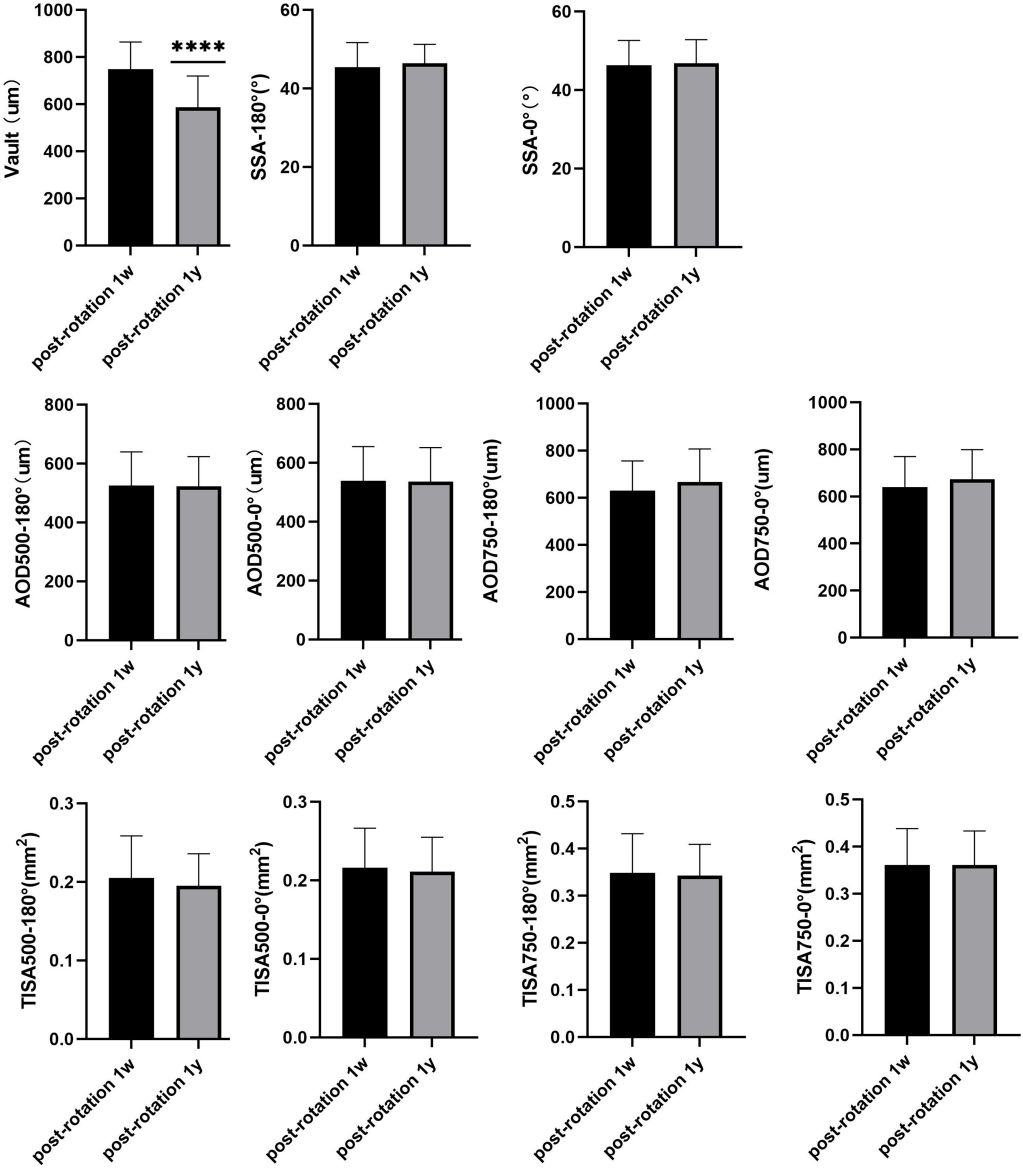
Although the mean vault value had changed significantly in the 1 year follow-up (*p* < 0.001), the SSA, AOD500, AOD750, TISA500, and TISA750 at 180° and 0° had remained well (all *p* > 0.05). **** means that the *p* < 0.001.

**Figure 5:**
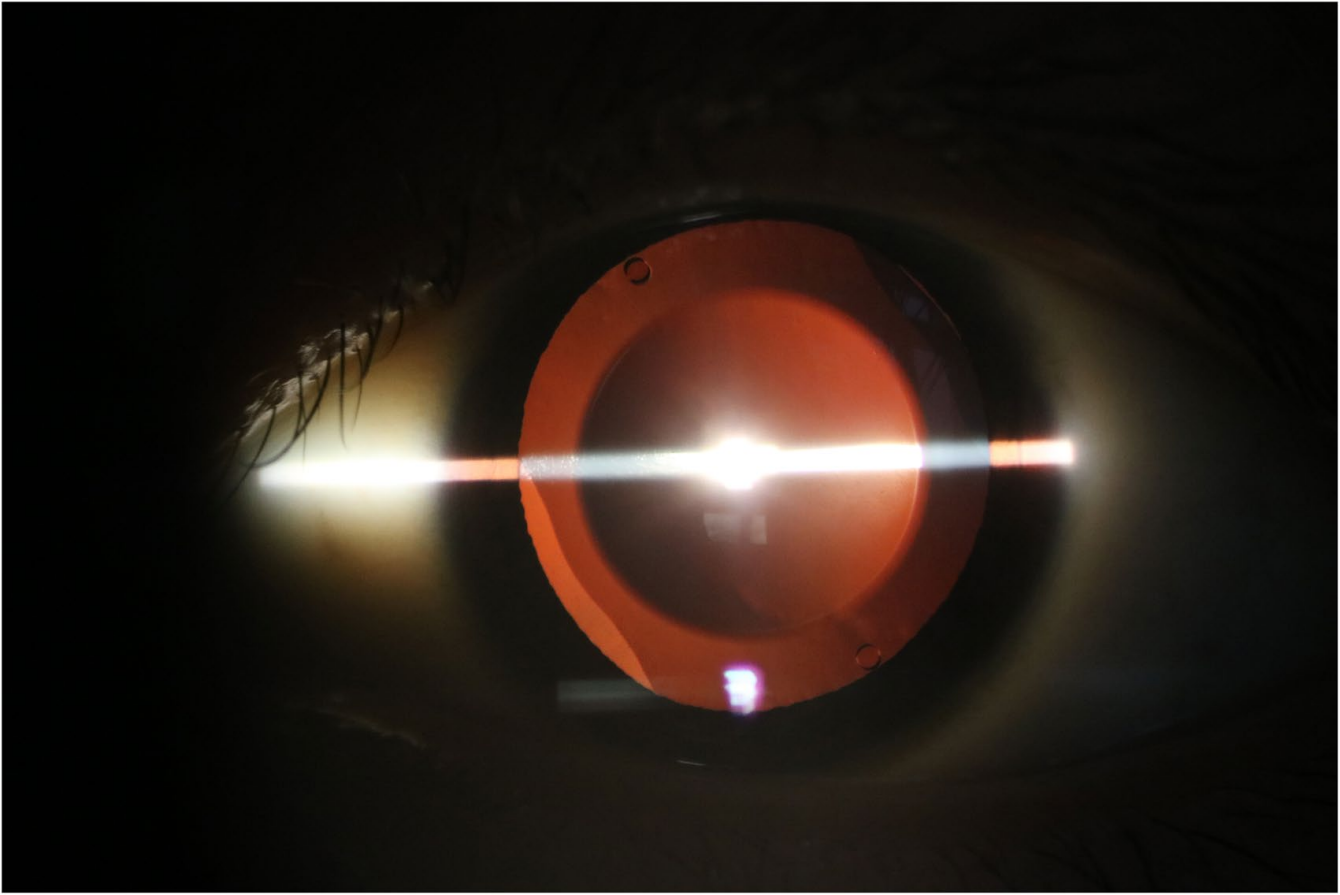
The implantable collamer lens position has remained well *in situ* for 2.5 years following rotation to the oblique position in patient C.

**Figure 6:**
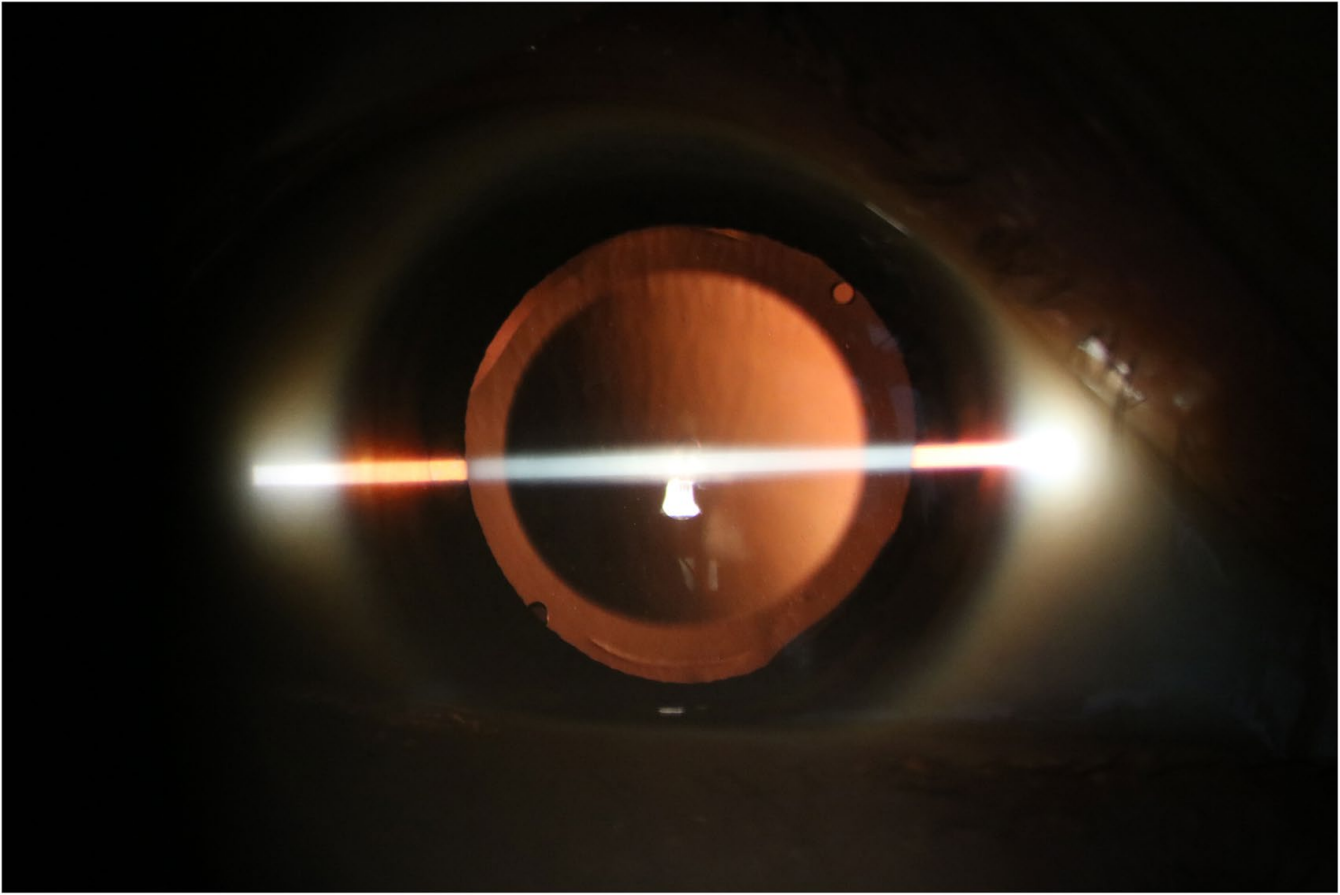
The implantable collamer lens position has remained well *in situ* for 3 years following rotation to the oblique position in patient D.

In total, 16 of 22 patients (72.72%) had completed the 1-year follow-up. There was no intraoperative or postoperative complication. The mean vault value had changed from 747.50 ± 116.07 μm 1-week post-rotation to 586.87 ± 132.65 μm (*p* < 0.001). However, the SSA, AOD500, AOD750, TISA500, and TISA750 at 180° and 0° had remained well as 45.43 ± 6.26° and 46.43 ± 4.81°, 46.29 ± 6.32° and 46.75 ± 6.06°, 525.56 ± 114.22 and 522.81 ± 100.93 μm, 538.00 ± 117.31 and 535.81 ± 115.91 μm, 630.43 ± 125.55 and 666.31 ± 140.75 μm, 640.25 ± 129.38 and 673.12 ± 125.91 μm, 0.205 ± 0.053 and 0.194 ± 0.041 mm^2^, 0.216 ± 0.050 and 0.210 ± 0.043 mm^2^, 0.348 ± 0.082 and 0.342 ± 0.066 mm^2^, 0.360 ± 0.076 and 0.361 ± 0.072 mm^2^ (all *p* > 0.05; Supplementary Table 3; Figure 4). ICL position was stable and stayed *in situ* (Figures 5, 6).

### Discussion

Implantable collamer lens implantation has become more prominent for the correction of myopia. There are multiple advantages including the relative maintenance of corneal integrity and the reversibility of the procedure. It has been demonstrated to be safe and effective (6, 7).

Implantable collamer lens sizing has traditionally been based on the white-to-white distance (7). There has been some uncertainty in determining the best size for ICLs. OCT and ultrasound biomicroscopy (UBM) have recently been utilized. However, inappropriate vault size still occurs likely secondary to anatomic and physiologic factors (8).

Acceptable post-operative vault values have been considered to be <750 μm (9). A low vault has been associated with increased risks of cataract development, while a high vault has been associated with pupil ovalization, iritis, pigment dispersion, angle crowding, liberation of inflammatory mediators, iris chaffing, endothelial cell loss, and increased risk of angle-closure glaucoma (1). Matarazzo et al. have observed mild/moderate anisocoria due to high ICL vault and they believe that anisocoria was caused by the large ICL which pushed the iris forward and increased the pupil size (4).

Implantable collamer lens sizing based on WTW distance and ACD, without UBM, was used in the FDA Visian study for myopia (10, 11). The resulting ICL vault, however, is difficult to predict precisely. Implantable Collamer Lens sizing using UBM-measured STS distance is supposed to be more accurate than the conventional WTW distance method because the ICL haptic footplates are located on the ciliary sulcus (12). Studies have shown that either OCT or UBM was not statistically greater than the other measurements (13). A meta-analysis showed that there was no clinically meaningful or statistically significant difference in WTW and sulcus-to-sulcus (STS) sizing methodologies in achieving an optimal vault (8). Gimbel et al. stated that they have not been able to avoid some high and some low vaulting in spite of using the UBM in partnership with the Orbscan II (14). Lee has used UBM to measure the STS for determining the proper ICL size; however, they stated that even now they are still achieving unexpected high or low vaulting (15). There are better methods for choosing ICL currently, although not definitive (16). However, ICL oversizing is still a problem.

We know that the relationship between the ICL size and horizontal sulcus-to-sulcus diameter (STS) determines the postoperative vault (1). The ciliary sulcus is likely vertically oval, which has been previously demonstrated using UBM (2, 17). In the observational study conducted by Oh et al. demonstrated that vertical diameters measured by UBM were longer than horizontal ones in all eyes examined. Vertical diameters were the longest among the four meridians, and the mean difference between vertical and horizontal diameters was 0.67 ± 0.26 mm (17). Similar results were found by Biermann et al. (2). They observed that vertical STS diameter was 0.35 ± 0.32 mm and 0.35 ± 0.16 longer in emmetropic and myopic eyes, respectively. Matarazzo et al. (4) in their case report showed that the angle-to-angle (ATA) diameter vertically was 0.579 mm larger than horizontally by CASIA AS-OCT, which guaranteed the feasibility of their rotation.

ICL rotation from horizontal to oblique is a novel method for reducing the high postoperative vault, which provides more reasonable expectations with a not too high or too low vault. It has been previously described in Matarazzo’s case report (4). Zaldivar et al. have reported their satisfactory results of ICL rotation under the guidance of intraoperative OCT (5).

Although the advantage of intraoperative high-resolution OCT systems is well-known and obvious, providing real-time quantitative measurements of surgical outcomes and, therefore, allowing critical decision-making in time, these systems are expensive and not all surgical centers can afford them. It also needs time before surgeons can use this equipment, while there are so many ICL implantation surgeries ongoing in the meantime. In addition, intraoperative OCT provides only a relatively narrow window and cannot show the full situation of the anterior chamber angle which is more relevant to the risk of postoperative angle-closure glaucoma. Third, real-time vaults provided by intraoperative OCT would be influenced by the factors during the operation such as perfusion pressure and visco-elastic. Therefore, although intraoperative OCT can show important real-time situations, it cannot provide a comprehensive image and an accurate postoperative vault.

Our research has first observed the effectiveness and stability of this novel surgical procedure to deal with high postoperative vault without the use of intraoperative OCT, which can instruct surgeons to deal with tricky postoperative high vault cases when they do not possess any intraoperative OCT system.

In our research, the vault had declined significantly 1-week post-rotation, and the SSA, AOD500, AOD750, TISA500, and TISA750 at 180° and 0° had increased significantly accordingly.

The SSA is the most intuitionistic parameter, while the AOD500, AOD750, TISA500, and TISA750 provide a more accurate change of the anterior chamber angle before and after the ICL rotation. The improvements of all these anterior chamber angle indices indicate that the risks of postoperative complications relative to the high vault can be reduced by ICL rotation. In our observation, we noticed that the mean vault value had declined in the 1-year follow-up. This situation has been described in several previous studies. Fernandez-Vega-Cueto et al. have reported that the mean postoperative vault varied from 409 ± 196 μm at 12 months to 349 ± 165 μm at 36 months (18). Guber et al. have reported that the vault measured a mean (SD) of 426 (344) um immediately postoperatively, decreasing to 213 (169) um at 10 years (6). The decrease of the vault in the long-term follow-up described in these studies was consistent with our observation. In the study by Zaldivar, they appealed to longer follow-up observation to see whether this vault change with time would also occur in ICL rotation cases (5). In our case series research, we have answered their question that this vault declining change with time does also occur in ICL rotation; however, it does not affect the structure of the anterior chamber angle (ACA) since there was no change in all the ACA indexes including SSA, AOD500, AOD750, TISA500, and TISA750 (all *p* > 0.05). The reason remains unclear, and it should be explored in the following study.

Our case series demonstrates the effectiveness of this novel surgical procedure. The rotation of a spherical ICL can reduce high postoperative vault effectively instead of an ICL exchange procedure which is of high cost and surgical risk. Rotation is much easier to be practiced than ICL exchange with less surgical injury. The novel surgical procedure has also shown excellent stability in our long-term follow-up which proved it a great alternative to the traditional ICL exchange. The limitation of this research is that the sample size is relatively small, and a more balanced gender distribution of subjects should be provided in the following study.

## Data availability statement

The raw data supporting the conclusions of this article will be made available by the authors, without undue reservation.

## Ethics statement

The studies involving human participants were reviewed and approved by the Institutional Ethics Committee of Chinese Academy of Medical Sciences. The patients/participants provided their written informed consent to participate in this study.

## Author contributions

YJ, YaL, and YiL were involved in the conception or design of the work, the acquisition, and analysis or interpretation of data for the work. YJ and TL drafting the work or revising it critically for important intellectual content. YJ, YaL, YiL, and TL final approval of the version to be published and agreement to be accountable for all aspects of the work in ensuring that questions related to the accuracy or integrity of any part of the work are appropriately investigated and resolved. All authors contributed to the article and approved the submitted version.

## Conflict of interest

The authors declare that the research was conducted in the absence of any commercial or financial relationships that could be construed as a potential conflict of interest.

## Publisher’s note

All claims expressed in this article are solely those of the authors and do not necessarily represent those of their affiliated organizations, or those of the publisher, the editors and the reviewers. Any product that may be evaluated in this article, or claim that may be made by its manufacturer, is not guaranteed or endorsed by the publisher.
