# Supplementary material for: The long-term observation of the rotation of implantable collamer lens as the management of high postoperative vault
Source: Front Med (Lausanne). 2023 Feb 23;10:1104047. doi: 10.3389/fmed.2023.1104047 (PMC9995363; doi:10.3389/fmed.2023.1104047)
Supplement: Supplementary file 1 [file Table_1.DOC]

**Supplemental Table 1. Descriptive statistics of the sample.**

|  | **Mean ± SD** | **Range** |
| --- | --- | --- |
| **Age (y)** | **28.68 ± 6.08** | **18 to 42** |
| **SE (D)** | **-9.63 ± 3.13** | **-3 to -16.75** |
| **ACD (mm)** | **3.29 ± 0.26** | **2.8 to 3.71** |
| **W to W (mm)** | **11.48 ± 0.43** | **10.7 to 12.3** |
| **ICL power (D)** | **-11.02 ± 3.12** | **-4.5 to -16.5** |
| **SD: standard deviation;** | | |
