# Supplementary material for: The long-term observation of the rotation of implantable collamer lens as the management of high postoperative vault
Source: Front Med (Lausanne). 2023 Feb 23;10:1104047. doi: 10.3389/fmed.2023.1104047 (PMC9995363; doi:10.3389/fmed.2023.1104047)
Supplement: Supplementary file 2 [file Table_2.DOC]

**Supplemental Table 2. The difference in all values pre-rotation and 1 week post-rotation**

|  | **Vault**  **（um）** | **SSA-180°**  **(°)** | **SSA-0°**  **(°)** | **AOD500-180°**  **（um）** | **AOD500-0°**  **（um）** | **AOD750-180°**  **（um）** | **AOD750-0°**  **（um）** | **TISA500-180°**  **(mm^2^)** | **TISA500-0°**  **(mm^2^)** | **TISA750-180°**  **(mm^2^)** | **TISA750-0°**  **(mm^2^)** |
| --- | --- | --- | --- | --- | --- | --- | --- | --- | --- | --- | --- |
| **Pre -**  **rotation** | **951.81±154.26** | **30.40±7.91** | **32.37±7.48** | **303.27±87.99** | **323.81±89.15** | **387.95±99.43** | **435.68±106.72** | **0.109±0.034** | **0.123±0.034** | **0.194±0.056** | **0.216±0.055** |
| **1w Post-**  **rotation** | **772.27±119.40** | **45.14±6.75** | **46.23±6.39** | **522.45±122.16** | **536.13±121.66** | **630.81±133.59** | **643.36±132.82** | **0.202±0.053** | **0.212±0.051** | **0.345±0.083** | **0.358±0.079** |
| **t** | **4.469** | **7.751** | **7.011** | **7.890** | **7.092** | **9.221** | **6.522** | **7.973** | **6.722** | **8.313** | **6.953** |
| ***p*** | **< 0.001*** | **< 0.001*** | **< 0.001*** | **< 0.001*** | **< 0.001*** | **< 0.001*** | **< 0.001*** | **< 0.001*** | **< 0.001*** | **< 0.001*** | **< 0.001*** |
