# Supplementary material for: The long-term observation of the rotation of implantable collamer lens as the management of high postoperative vault
Source: Front Med (Lausanne). 2023 Feb 23;10:1104047. doi: 10.3389/fmed.2023.1104047 (PMC9995363; doi:10.3389/fmed.2023.1104047)
Supplement: Supplementary file 3 [file Table_3.DOC]

**Supplemental Table 3. The difference in all values 1 week post-rotation and at least 1 year follow-up**

|  | **Vault**  **（um）** | **SSA-180°**  **(°)** | **SSA-0°**  **(°)** | **AOD500-180°**  **（um）** | **AOD500-0°**  **（um）** | **AOD750-180°**  **（um）** | **AOD750-0°**  **（um）** | **TISA500-180°**  **(mm^2^)** | **TISA500-0°**  **(mm^2^)** | **TISA750-180°**  **(mm^2^)** | **TISA750-0°**  **(mm^2^)** |
| --- | --- | --- | --- | --- | --- | --- | --- | --- | --- | --- | --- |
| **1w Post-**  **rotation** | **747.50±116.07** | **45.43±6.26** | **46.29±6.32** | **525.56±114.22** | **538.00±117.31** | **630.43±125.55** | **640.25±129.38** | **0.205±0.053** | **0.216±0.050** | **0.348±0.082** | **0.360±0.076** |
| **1y Post-**  **rotation** | **586.87±132.65** | **46.43±4.81** | **46.75±6.06** | **522.81±100.93** | **535.81±115.91** | **666.31±140.75** | **673.12±125.91** | **0.194±0.041** | **0.210±0.043** | **0.342±0.066** | **0.361±0.072** |
| **t** | **5.591** | **1.083** | **0.322** | **0.118** | **0.082** | **1.000** | **1.183** | **0.906** | **0.492** | **0.400** | **0.004** |
| ***p*** | **< 0.001*** | **0.296** | **0.752** | **0.908** | **0.936** | **0.333** | **0.255** | **0.379** | **0.630** | **0.695** | **0.997** |
